# Global Consultation for Clinical Xenotransplantation: International Xenotransplantation Association Consensus and Communiqué Update

**DOI:** 10.1111/xen.70157

**Published:** 2026-08-03

**Authors:** Wayne J. Hawthorne, Eckhard Wolf, Peter J. Cowan, Wei Wang, Ik Jin Yun, Paolo Brenner, Greg Korbutt, Raphael P. H. Meier, Megan Sykes, Léo H. Bühler, Linda Scobie, Olga Garkavenko, Hyunil Kim, Daniel J. Hurst, Richard N. Pierson III, Robert Rieben, Manuel Pascual, Nicole Scholes‐Robertson, Emanuele Cozzi, Adrián Abalovich, Alexandre Loupy, Hidetaka Hara, Rita Bottino, Burcin Ekser, Muhammad M. Mohiuddin, Jay Fishman

**Affiliations:** ^1^ Department of Surgery Westmead Hospital Westmead Institute for Medical Research Faculty of Medicine and Health University of Sydney Westmead NSW Australia; ^2^ Gene Center Ludwig Maximilian University of Munich (LMU) Munich Germany; ^3^ Immunology Research Centre St Vincent's Hospital University of Melbourne Melbourne Australia; ^4^ Engineering Center for Xenotransplantation The Third Xiangya Hospital Central‐South University Changsha China; ^5^ Department of Surgery Konkuk University Hospital Konkuk University Seoul South‐Korea; ^6^ Department of Cardiac Surgery Klinikum Großhadern Ludwig Maximilian University of Munich (LMU) Munich Germany; ^7^ Department of Surgery University of Alberta Edmonton AB Canada; ^8^ Department of Surgery University of Maryland School of Medicine Baltimore Maryland USA; ^9^ Columbia Center for Translational Immunology Columbia University College of Physicians and Surgeons Columbia University New York New York USA; ^10^ Cantonal Hospital Fribourg Faculty of Science and Medicine University of Fribourg Fribourg Switzerland; ^11^ Department of Biological and Biomedical Sciences School of Health and Life Sciences Glasgow Caledonian University Glasgow Scotland UK; ^12^ NZeno Limited Auckland New Zealand; ^13^ Optipharm, Inc. Cheongju South Korea; ^14^ Department of Medical Education & Scholarship Rowan‐Virtua School of Osteopathic Medicine Stratford New Jersey USA; ^15^ Division of Cardiac Surgery and Center for Transplantation Sciences Department of Surgery Massachusetts General Hospital Boston Massachusetts USA; ^16^ Department For BioMedical Research (DBMR) University of Bern Bern Switzerland; ^17^ Transplantation Center Department of Surgery Lausanne University Hospital and University of Lausanne Lausanne Switzerland; ^18^ Sydney School of Public Health Faculty of Medicine and Health University of Sydney Westmead NSW Australia; ^19^ Transplant Immunology Unit Padua University Hospital Padua Italy; ^20^ Eva Perón Hospital of San Martin Buenos Aires Argentina; ^21^ Department of Kidney Transplantation Assistance Publique ‐ Hôpitaux de Paris Université Paris Cité Paris Institute for Transplantation and Organ Regeneration Paris France; ^22^ The Transplantation Institute of the Second Affiliated Hospital Hainan Medical University Hainan China; ^23^ Imagine Islet Center Imagine Pharma Pittsburgh Pennsylvania USA; ^24^ Division of Intraabdominal Transplant Department of Surgery Stritch School of Medicine Loyola University Medical Center Loyola University Chicago Maywood Illinois USA; ^25^ Department of Surgery Cardiac Xenotransplantation Program University of Maryland School of Medicine Baltimore Maryland USA; ^26^ Harvard Medical School Transplant Infectious Disease and Compromised Host Program and Transplant Center Massachusetts General Hospital Boston Massachusetts USA

**Keywords:** Changsha Communiqué, ethical oversight, equity, global governance, international guidelines, legislation, long‐term surveillance, regulatory frameworks, transplantation, World Health Organization (WHO), xenotransplantation

## Abstract

Xenotransplantation has entered a phase of accelerated clinical translation, necessitating renewed international consensus on governance, ethics, safety, and regulatory oversight. In September 2025, the International Xenotransplantation Association (IXA), in partnership with The Transplantation Society (TTS) and with engagement from the World Health Organization (WHO), convened a Global Consultation in Geneva to review, update, and harmonise international guidance for clinical xenotransplantation. This IXA consultation marked the twentieth anniversary of the first WHO Xenotransplantation Advisory Consultation in 2005 and built upon the foundational principles established through the Changsha Communiqué (2008, 2011 and 2018) and subsequent IXA and WHO‐led initiatives. Responding to rapid scientific advances, including some defined donor genetic standards, improved immunosuppression, enhanced biosecurity, and strengthened infectious disease surveillance, the IXA undertook an 18‑month, structured, evidence‑based revision process. Seven expert working groups, comprising approximately forty internationally recognised specialists from fourteen countries, reviewed developments across source animal standards and biosecurity, clinical trial design and oversight, immunosuppression and patient management, infectious disease risk and surveillance, ethical and legal frameworks, international governance, and emerging technologies. Draft recommendations were further refined through iterative consultation and plenary deliberation at the in‑person meeting held in Geneva, September 2025. The consultation proposed updated Principles and Recommendations directed to WHO, national regulators, investigators and sponsors, and the IXA/TTS communities. Key elements include proportionate risk benefit assessments, robust donor and recipient surveillance, long‑term biobanking and monitoring, transparency and public engagement, harmonised regulatory oversight consistent with existing allotransplantation frameworks, and equitable access to future clinical applications. The revised guidance aligns with contemporary regulatory expectations of major international jurisdictions regulatory authorities including (AEMPS, ANMAT, CONABIA, CONFEPRIS, EMA, FDA, GTAC, HC, IEC, INCUCAI, Medsafe, MFDS, MHRA, MHLW, NMPA, NZGTAC, MFDS, MHRA, TGA); and consolidates international consensus at a critical juncture for the field. This IXA Global Consultation provides an authoritative, forward‑looking framework to support safe, ethical, and globally coordinated clinical xenotransplantation as it transitions from experimental innovation to regulated clinical practice.

## 1 Background and Introduction

The International Xenotransplantation Association (IXA) was formed by leading experts in the field of transplantation as the world's leading organisation dedicated to advancing xenotransplantation science, ethics, and clinical translation. Established as the pre‐eminent global authority in the field, IXA has led international efforts to ensure that xenotransplantation has developed safely, responsibly, and in alignment with evolving scientific and societal expectations [1]. Established at The Transplantation Society (TTS) Congress in Montreal in 1998 as the first association formed under the auspices of TTS, IXA continues to be guided by the founding principles rooted in the Cartagena Protocol on Biological Diversity [2, 3], promoting the safe use of genetically modified organisms to enhance human welfare while protecting biological diversity and care [4].

From these beginnings almost three decades ago, IXA has worked with TTS and the World Health Organisation (WHO) to establish standards for xenotransplantation research and translation to the clinic. The IXA has played a key role in shaping the ethical, scientific, and regulatory foundations for clinical xenotransplantation, working in close alignment with international organisations, national authorities, and professional societies. These principles have been a major influence in framing subsequent international policy discussions. In 2001, WHO published the ‘Guidance on Xenogeneic Infection/Disease Surveillance and Response: A Strategy for International Cooperation and Coordination’, addressing the global infectious risks associated with xenogeneic procedures [5]. In 2003, IXA outlined ethical and governance requirements needed for responsible clinical translation, including the need for robust preclinical evidence, effective regulatory oversight by competent authorities, and formal approval by institutional bodies responsible for human research ethics and animal welfare and published the first in a series of “Guidelines” on the topic [6, 7].

At the 57th World Health Assembly in 2004, resolution WHA57.18 was adopted and urged Member States “to allow xenogeneic transplantation only when effective national regulatory control and surveillance mechanisms overseen by national health authorities are in place” [8]. The alignment between this resolution and prior IXA ethical guidance underscored the Association's formative influence on emerging international regulatory expectations. Resolution WHA57.18 was the catalyst for four WHO‐supported international meetings informed by expert input from the IXA council. The first Xenotransplantation Advisory Consultation was convened at WHO Headquarters, Geneva in 2005 [9]. Subsequently, the first WHO Global Consultation on Regulatory Requirements for Xenotransplantation Clinical Trials was held in Changsha, China. The meeting's consensus recommendations were published called the “Changsha Communiqué” [10, 11]. A second Global Consultation was held at WHO headquarters in Geneva in 2011, with the specific focus on infectious risks associated with xenotransplantation, resulting in further guidance documents and the first outline for approaches to disease surveillance in individual recipients of nonhuman tissues [12, 13].

As xenotransplantation technologies advanced rapidly over the subsequent decade, the need to reassess and update international guidance became evident. To mark the tenth anniversary of the WHO Changsha Communiqué and to formally revisit the Principles and Recommendations directed toward investigators, regulators, Member States, and international organizations, the Third WHO Global Consultation on Regulatory Requirements for Xenotransplantation Clinical Trials was convened. Jointly organised by the IXA and TTS and with support from the WHO, the meeting was held in Changsha, Hunan Province, China, in December 2018 [14].

This meeting report summarises the latest global consultation held in Geneva, September 2025. To commemorate the twentieth anniversary of the original 2005 WHO Xenotransplantation Advisory Consultation, IXA, in conjunction with TTS, convened a global consultation to review prior guidance documents, examine progress in regulatory practice, and to update recommendations for clinical xenotransplantation in the context of ongoing scientific, clinical, and governance developments. This consultation aligned with both the 18th Congress of the IXA and the WHO consultation on regulatory aspects of xenotransplantation, thereby incorporating the continuity of international collaboration and sustained engagement that has shaped the governance of global xenotransplantation over the past two decades.

This update and revision consolidate key developments that have emerged since the previous global consultation, developing a document that reflects global standards, regulatory expectations, and scientific advances. Beginning 18‐months ahead of the final in‐person‐meeting, the IXA undertook a structured, evidence‐based process to ensure alignment with international best practices. Seven expert working groups reviewed and updated the content spanning developments since the 2018 revision, culminating in a consultative meeting at which the collective findings were presented, discussed and debated, and finally integrated into this current update. Some 40 international subject‐matter experts from 14 countries were invited to contribute to this critical revision. Their collective input was sought to ensure that this updated report of the Communiqué remains a globally authoritative and forward‐looking resource for the international community.

### 1.1 Overview of Groups and Their Tasks

**FIGURE 1:**
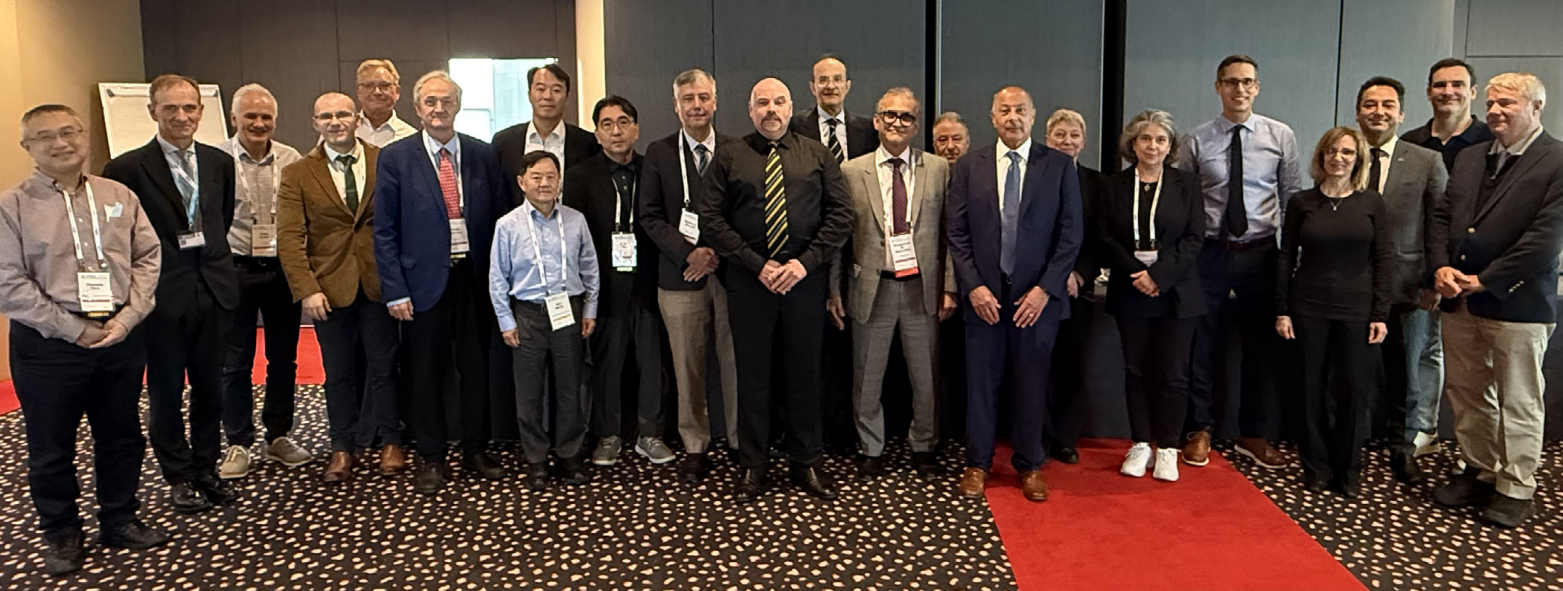
Photograph at the end of the meeting of most of the delegates who attended‐in‐person at the Global Consultation, Geneva, Switzerland, September 2025. **Standing Left to Right** Hidetaka Hara, China; Léo H. Bühler, Switzerland; Peter J. Cowan, Australia; Daniel Hurst, USA; Ralf R. Tönjes, Germany; Paolo Brenner, Germany; Hyunil Kim, South Korea; Wei Wang, China; Ik Jin Yun, South‐Korea; Manuel Pascual, Switzerland; Wayne J. Hawthorne, Australia; Emanuele Cozzi, Italy; Muhammad M. Mohiuddin, USA; Adrian Abalovich, Argentina; Jay Fishman, USA; Olga Garkavenko, New Zealand; Antonia Godehardt, Germany; Raphael Meier, Switzerland; Rita Bottino, USA; Burcin Ekser, USA; Alex Loupy, France; Richard N. Pierson III, USA.

Commissioned by the IXA Council, the meeting was convened and coordinated by Wayne Hawthorne, who provided strategic leadership aligned with the 2018 Communique’ revision process. His responsibilities encompassed defining the scope and objectives of the meeting, establishing and coordinating the working group structure, guiding and moderating the plenary deliberations, and overseeing development and completion of the final summary report. The convenor worked in close consultation with the appointed rapporteurs, working group members, and the IXA Council and ethics committee to ensure balanced representation, scientific rigor, and alignment with international standards and governance frameworks. As can be seen in Figure 1, a photograph was taken at the conclusion of the global consultation capturing most of the remaining delegates who attended the in person section of the meeting in Geneva, Switzerland, September 2025.

### 1.2 Meeting Structure and Working Groups

To facilitate focused and in‐depth discussion, participants were assigned to one‐of‐seven thematic working groups, each addressing a critical domain in xenotransplantation requiring review. Each group was chaired by a designated rapporteur and composed of internationally recognised experts with complementary expertise but was also able to draw from the other groups to integrate and ensure complementarity from all topics and groups. Although working groups met independently prior to the meeting to develop draft positions, all work was reviewed and subsequently presented, discussed, and refined by all participants prior to and at the in‐person‐meeting. The structure and remit of the working groups is outlined below.

**Working Group 1:** Source Animal Standards and Biosecurity

**Focus:** The need for closed‐herd management, biosecurity systems, genetic modification strategies, donor animal selection criteria, facility standards, and microbiological safety related to donor breeding programs. Emphasis was placed on strategies to reduce infectious risks and enhance graft safety.

**Rapporteur**: Eckhard Wolf

**Final Working Group Members: **Peter Cowan, Wei Wang, Ik Jin Yun

**Working Group 2:** Clinical Trial Design and Oversight

Focus: Selection and review of appropriate clinical trial protocols, regarding the various organs, tissues and cells to be transplanted, regulatory and ethical compliance, patient selection criteria, trial conduct and monitoring, and interaction with regulatory authorities. **Rapporteur**: Muhammad Mohiuddin

**Final Working Group Members: **Paolo Brenner, Greg Korbutt, Ralf R. Tönjes (Observer status)

**Working Group 3:** Immunosuppression, Tolerance Induction, and Patient Management Focus: The development and optimisation of the suitable immunosuppressive regimens, organ, tissue and cell‐specific approaches, adaptation to different genetically modified donor animals, and emerging strategies for localised or graft delivered immunomodulation. **Rapporteur**: Raphael Meier

**Final Working Group Members: **Megan Sykes, Burcin Ekser, Leo Bühler

**Working Group 4: Infectious Disease Risk and Surveillance**

**Focus**: To ensure the minimisation of potential Xenozoonotic risks, donor and recipient health screening, long‐term monitoring of recipients and close contacts, sample archiving, public health safeguards, and pathogen‐specific issues including porcine endogenous retroviruses (PERVs) and porcine cytomegalovirus (PCMV). Advanced detection strategies, such as metagenomic sequencing and tailored antimicrobial prophylaxis.

**Rapporteur**: Jay Fishman

**Final Working Group Members: **Linda Scobie, Olga Garkavenko, Hyunil Kim

**Working Group 5: Ethical and Legal Frameworks**

**Focus**: To review ethical criteria for recipient selection, informed consent processes, evolving national and international legislative frameworks, strategies for public and patient engagement, need for engagement and views of religious denominations.

**Rapporteur**: Daniel Hurst

**Final Working Group Members: **Richard (Robin) Pierson, Robert Rieben, Manuel Pascual, Nicole Scholes‐Robertson

**Working Group 6: International Collaboration and Governance**

**Focus**: Define the roles of national and international agencies, professional societies, and regulatory authorities in the development, implementation, and harmonisation of xenotransplantation guidance and oversight frameworks worldwide in relation to the international guidance.

**Rapporteur**: Emanuele Cozzi

**Final Working Group Members: **Adrian Abalovich, Antonia Godehardt (Observer status)

**Working Group 7: New Technologies for Xenotransplantation**

**Focus**: Provide the scope for the application of emerging technologies to donor and recipient selection, graft monitoring, and functional assessment, including artificial intelligence driven clinical decision tools, multiomics approaches (genomics, proteomics, metabolomics), circulating biomarkers such as donor‐derived cell‐free DNA and cytokine profiling, and advanced tools for immune and infection surveillance.

**Rapporteur:** Alex Loupy

**Final working group members**: Rita Bottino, Hidetaka Hara

## 2 Basis for Recommendations as Assigned by the IXA and Expert Panels

### 2.1 Working Group 1: Source Animal Standards and Biosecurity

The overview presented by the group acknowledged that xenotransplantation has progressed from a predominantly experimental discipline to one entering early‑phase clinical application. Central to this transition has been the development of genetically engineered porcine source animals capable of addressing well‑defined immunological and physiological incompatibilities with human recipients. Advances in gene‑editing technologies have enabled precise and reproducible modification of key donor traits, including elimination of dominant carbohydrate xenoantigens and introduction of human genes conferring protection against complement activation, coagulation dysregulation, innate immune injury, and inflammatory responses.

Pigs, despite their phylogenetic distance from humans, are the preferred source of cells, tissues, and organs for xenotransplantation. Their advantages include rapid reproduction, suitability for pathogen‐free breeding, size‐matching for human recipients, ethical acceptability, and the availability of well‐established tools for precise genetic modification [15].

Importantly, the evidence reviewed by the group indicates that clinical translation is being driven not so much by the ever‑increasing number of genetic modifications, but rather by the identification of a core set of essential modifications that are biologically effective, consistently expressed, and compatible with scalable production of source animals [16, 17]. Excessive or poorly integrated or poorly characterized genetic edits may introduce complexity in breeding, raise concerns regarding undesired transgene interactions and variability in tissue expression, and may complicate regulatory oversight. The expert group concurred that the selection of genetically modified source pigs for clinical use should be guided by demonstrated functional necessity and reliability, rather than maximal technical feasibility [15].

### 2.2 Immune and Physiological Barriers: Progress and Residual Uncertainty

Evidence demonstrates substantial progress in preventing hyperacute rejection, mitigating subsequent antibody‑mediated injury, and thrombotic microangiopathy, historically the principal barriers to xenograft survival. The elimination of major carbohydrate antigens combined with expression of human complement and coagulation regulatory proteins now represents a widely accepted baseline platform for clinical consideration regardless of the organ to be transplanted [18, 19, 20].

Nevertheless, xenograft rejection remains multifactorial and context‑dependent. Variability in antibody responses, innate immune activation, inflammatory signalling, and coagulation compatibility continues to influence outcomes, even with advanced donor genotypes. These findings reaffirm a central premise of the Changsha Communiqué: that clinical xenotransplantation must proceed incrementally, with rigorous scientific justification, comprehensive monitoring, continued evaluation of combinations of transgenic modification of donor source animals and the capacity for regulatory oversight to assess donor animals for suitability including the capacity for pathogen surveillance [16, 17].

### 2.3 Source Animal Selection, Growth Control, and Biosafety

Consistent with the Changsha Communiqué principles addressing public health protection and biosafety, the group underscored the critical importance of donor pig selection beyond genetic modifications. Control of post‑transplant organ growth, appropriate size matching, and genetic background homogeneity were identified as key determinants of graft performance, particularly in life‑supporting organ xenotransplantation. To limit excessive donor pig and organ growth, knockout of the growth hormone receptor (GHR) gene has been implemented [21], reducing body size by ∼50% with a risk for undesired obesity and metabolic changes [22, 23]. An acceptable option may be to use naturally smaller breeds.

Equally, the group reaffirmed that pathogen surveillance and exclusion, most notably with respect to porcine cytomegalovirus and porcine endogenous retroviruses, remain essential requirements for clinical translation. Emerging mitigation strategies, including targeted viral inactivation [24, 25] and stringent source‑animal screening, are available. These conditions complement the need for long‑term recipient monitoring and transparent international reporting, consistent with the mandates of the Changsha Communiqué.

### 2.4 Clinical Experience in Cardiac and Renal Xenotransplantation

The group reviewed the growing body of preclinical and early clinical experience in cardiac and renal xenotransplantation. These data demonstrate that genetically modified porcine organs can successfully function in primate and human recipients for clinically meaningful periods [26, 27, 28]. Variable outcomes in first‑in‑human cases, including graft failure related to immune injury, complement and coagulation disturbances, infections and varied immunosuppression management, confirm that xenotransplantation remains a complex, investigational intervention.

The group emphasised that these early clinical cases represent appropriate application of the Changsha framework: cautious, closely supervised clinical exploration informed by robust preclinical data. This is seen to reinforce the necessity for harmonised trial design, standardised immunological assessment, and international data sharing to refine practice and avoid unnecessary duplication of risk.

### 2.5 Summary Recommendations

- Recognition of organ‑specific risk profiles and genetic requirements;
- Clear articulation of minimal rather than maximal donor genetic modification standards;
- Continued prioritisation of biosafety, transparency, and long‑term microbiological surveillance;
- Continued support for undertaking preclinical studies of new donor source animal genetic modifications and new combinations of immune suppressive strategies;
- Explicit acknowledgement of staged clinical introduction as an expected and appropriate pathway.

## 3 Working Group 2: Clinical Trial Design and Oversight

The group's deliberations recognised that, following several decades of rigorous preclinical investigation, xenotransplantation has entered a phase of cautious clinical application. Recent first‑in‑human cardiac and renal xenotransplants, together with emerging auxiliary liver xenotransplant studies, provide early evidence of feasibility while simultaneously underscoring the ethical, regulatory, and scientific requirements that must guide future clinical trials. The group synthesised lessons from relevant large‑animal models and early human experience to propose updates for structured frameworks for patient selection, trial design, and clinical oversight.

A central conclusion was that early‑phase xenotransplantation trials must prioritise patient safety, scientific validity, and ethical integrity over comparative efficacy. Candidate selection should focus on individuals with life‑threatening disease for whom no timely or acceptable allotransplant or alternative therapy exists, and who demonstrate sufficient psychosocial stability and support to comply with prolonged follow‑up and monitoring obligations. Consistent with existing regulatory guidance, source‑animal microbiological screening, microbial surveillance including for unknown human pathogens, and predefined stopping rules were regarded as integral components of trial design rather than adjunct considerations.

It was suggested that future liver xenografts or pig‐to‐human extracorporeal liver perfusion, positioned as temporary bridges to recovery or subsequent allotransplantation, demonstrate a uniquely tailored xenotransplant application that requires a unique set of patient selection criteria and trial designs.

The next generation of xenotransplantation clinical trials should emphasize standardization of patient selection, immunologic monitoring, with deliberate ethical patient selection governed by consensus amongst scientists and clinical trialists.

### 3.1 Lessons From Early Human and Preclinical Experience

The group reviewed the cumulative experience from compassionate‑use authorisations and decedent‑model studies involving cardiac, renal, and hepatic xenotransplantation. First‑in‑human porcine heart xenotransplants, early living‑recipient porcine kidney transplants, emerging auxiliary liver support studies, and decedent model investigations collectively demonstrate the biological feasibility and functional capability across organ systems and the utility of decedent models of clinical xenotransplantation. These experiences also highlight the importance of rigorous trial standardisation as clinical activity expands.

Key reproducible lessons from preclinical and early clinical cardiac and renal xenotransplantation include:

1. Donor pathogen control—stringent screening and exclusion of donor swine carrying porcine cytomegalovirus (PCMV/PRV) and other relevant pathogens are essential determinants of graft performance and recipient safety;
2. Humoral mechanisms—non‑Gal antibodies remain clinically relevant mediators of graft injury, even in advanced genetically modified donor platforms including α‐Gal–null donors;
3. Immunosuppressive strategy—costimulation blockade‑based regimen (e.g. anti‐CD40/154), combined with conventional agents (e.g. Tacrolimus, MMF, steroids) remain central to graft protection, although recent clinical studies suggest alternative approaches warrant further evaluation;
4. Adaptive trial oversight – trials should use predefined safety endpoints, microbiological standards, and hematologic parameters to guide escalation, modification, or early termination of trials. Additional surveillance and ethical principles should be standardized.

### 3.2 Organ‑Specific Considerations

#### 3.2.1 Kidney Xenotransplantation

Kidney xenotransplantation has emerged as the most advanced candidate for structured early‑phase clinical trials, including recent FDA approval for several limited prospective studies. Specific candidates include adults with end‑stage renal disease who face prohibitive allograft wait times (most likely to die or remain on dialysis with significant dialysis‑related morbidity rather than to receive an allotransplant), or high sensitisation. Exclusion criteria should address potential of uncontrolled infection, thrombotic risk, or inability to adhere to study requirements and intensive monitoring.

Primary trial endpoints should emphasise patient and graft survival and dialysis independence, with secondary endpoints including serial biopsy‑based immunologic assessment, renal functional trajectories, infectious burden, hospital utilisation, and patient‑reported outcomes. Early compassionate‑use experiences demonstrating immediate graft function and sustained dialysis independence reinforce the feasibility of carefully selected renal xenotransplantation recipients under structured oversight. The MGH compassionate‐use cases, demonstrated immediate graft function and safe discharge from hospital without xenozoonotic complications [29].

#### 3.2.2 Heart Xenotransplantation

Cardiac xenotransplantation remains appropriate for adult patients with advanced heart failure who are ineligible for durable mechanical circulatory support or allotransplantation and who face high predicted morbidity or mortality without transplantation [30].

Primary endpoints should include survival and freedom from mechanical support, with secondary endpoints encompassing histologic rejection assessment (serial endomyocardial biopsies to determine rejection‐free‐interval), cardiac imaging (transthoracic echocardiography), functional status, infection surveillance, and patient reported quality‑of‑life measures.

The Maryland compassionate‐use clinical experience highlighted the critical importance of specified pathogen‑free donor animals and for durable graft performance and the need for screening of IVIG and blood products for porcine‐avid antibodies [27, 31]. These observations reinforce the need for rigorous donor screening and careful selection of adjunctive therapies and consideration to screen for PCMV and blood products or therapeutics against anti‐porcine antibodies.

#### 3.2.3 Liver Xenotransplantation

Porcine liver xenotransplantation is currently positioned as an auxiliary or bridging therapy using pig‐to‐human extracorporeal liver perfusion in acute hepatic failure, rather than definitive replacement.

Suitable candidates include patients with severe metabolic decompensation and limited survival prospects, but sufficient physiologic reserve to tolerate temporary xenogeneic support while awaiting recovery or subsequent allotransplantation.

Primary endpoints should focus on survival to recovery or allotransplantation, with secondary endpoints being biochemical stabilisation, hepatic encephalopathy severity, inflammatory parameters, and intensive care utilisation.

Unique challenges including coagulopathy, thrombocytopenia, and inflammatory dysregulation require organ‑specific trial designs. Preclinical and early clinical data demonstrate transient platelet consumption which is generally transient. A genetically modified liver xenograft can function for up to 30 days, giving credence to its ability to bridge to allotransplantation or recovery [32, 33]. The first FDA trial for extracorporeal circulation of a porcine liver has now been approved [34].

### 3.3 Ethical and Regulatory Framework

The group re‐affirmed the views of Working Groups 3 and 5 that early xenotransplantation trials should adopt single‑arm, open‑label phase 1/2 designs emphasising safety, feasibility, and biological insight. Independent data safety monitoring boards must retain authority to pause or terminate enrolment at predefined safety thresholds.

The group also agreed with Working Groups 1 and 4 that regulatory oversight must remain centred on international guidance providing for national authorities to mandate designated pathogen‑free source herds, routine virological surveillance (with a focus on PERV and PCMV, and lifetime monitoring of recipients and close contacts [14, 35, 36].

In parallel, evolving ethical standards increasingly support moving beyond a strict “last‑resort” paradigm toward carefully justified early access based on clear unmet clinical need, capacity for benefit, informed consent (including comprehension of xenozoonotic risk), and psychosocial stability to sustain lifelong surveillance. The group agreed with Working Group 5's views on emphasised importance for embedding educational resources, long‑term follow‑up infrastructure, and equity considerations within trial budgets to ensure fair access and sustained compliance.

### 3.4 Summary Recommendations

- Clinical xenotransplantation has advanced to early‑phase human trials supported by robust preclinical evidence;
- Patient selection must balance unmet clinical need, anticipated benefit, and capacity for long‑term surveillance;
- Trial design should prioritise safety, standardisation, and adaptive oversight informed by early lessons;
- Organ‑specific biological and ethical considerations necessitate tailored trial frameworks;
- Harmonised regulatory and ethical governance remains essential to responsible clinical translation.

## 4 Working Group 3: Immunosuppression, Tolerance Induction, and Patient Management

The group acknowledged that, since the 2018 Changsha Communiqué, the clinical landscape of xenotransplantation had advanced significantly. Sustained function of pig‑to‑human kidney xenografts beyond six months [37], together with regulatory approval of early‑phase clinical trials, underscored the need to refine and update IXA guidance with a major focus on immunosuppressive strategies, tolerance‑oriented approaches, and comprehensive patient management. These developments reflect the maturation of the field from exclusively preclinical optimisation toward cautiously structured clinical implementation requiring urgent update of the earlier IXA's guidance [38].

### 4.1 Systemic Immunosuppression

Current immunosuppression protocols for xenotransplantation continue to rely on multidrug regimens adapted from allotransplantation but modified to address heightened xenoimmune responses. The basal calcineurin inhibitors, mycophenolate mofetil, and corticosteroids remain foundational elements of most clinical protocols, but are increasingly complemented or partially replaced by costimulation blockade using anti‑CD40 or anti‑CD154 monoclonal antibodies [39]. In preclinical kidney xenotransplantation models, such regimens have consistently outperformed calcineurin‑inhibitor‐dominant approaches, enabling life‑supporting graft survival exceeding one year and, in some programs, up to two years [6, 40]. Early clinical experiences have translated these principles into immediate graft function, with prevention of hyperacute rejection, and treatable episodes of acute cellular rejection [27, 29].

The group also highlighted the growing role of complement modulation in early post‑transplant management. Short‑term inhibition of C1, C5 or C3 has demonstrated utility in attenuating antibody‑mediated injury in both renal and cardiac xenotransplantation models. While complement inhibitors are increasingly incorporated into clinical protocols, the panel emphasised that endorsement of specific agents should remain flexible as newer drug classes, including FcRn inhibitors and additional innate immune modulators, approach clinical availability. In particular, next‐generation non‐thrombogenic anti‐CD154 agents have matured in preclinical and early clinical xenotransplantation settings and, together with anti‐CD40 monoclonal antibodies, are currently identified as the preferred co‐stimulation blockade platform for initial clinical trials.

### 4.2 Organ‑ and Donor‑Specific Variation

The expert panel underscored that no single immunosuppressive regimen was universally applicable across xenotransplantation modalities. Optimal strategies vary according to organ type, tissue or cell, along with the physiologic demands, and the genetic design of the donor animal. Kidney xenografts derived from triple‑gene knockout pigs expressing multiple human complement‑ and coagulation‑regulatory transgenes may permit less intensive immunosuppression than earlier donor platforms, although this remains incompletely validated in relevant models [41]. Of note, the impact of the third (CMAH) knockout on immunosuppressive requirements cannot be meaningfully assessed in Old World NHPs, which lack Neu5Gc and generate antibodies to new epitopes revealed by the CMAH knockout (the so‐called “fourth antigen” problem). This species‐specific limitation underscores the importance of carefully monitored human translation to evaluate TKO donor platforms. By contrast, cardiac xenotransplantation continues to require more aggressive control of humoral immunity, complement activation, and coagulation incompatibility [42].

Other applications present unique challenges. Porcine liver xenografts, currently evaluated primarily as temporary bridge therapies, are associated with coagulopathy, inflammatory activation, and metabolic adaptation. Lung xenotransplantation remains highly experimental, with limited survival reported in preclinical studies. Cellular therapies, including pancreatic islets [43] and hepatocytes [44], interact differently with innate and adaptive immune pathways and may benefit from immunosuppressive strategies that place greater emphasis on local immune modulation rather than systemic calcineurin inhibition.

### 4.3 Impact of Donor Genetic Engineering

Advances in donor engineering have transformed the immunologic landscape of xenotransplantation, progressing from single‑gene knockouts to complex donor platforms combining removal of major carbohydrate xenoantigens with multiple protective human transgenes. While these modifications have reduced natural antibody binding and early vascular injury in vitro and in limited in vivo settings, their individual and combined contributions require dissection in nonhuman primate models. Species‑specific differences in glycan recognition further limit the predictive value of these models, reinforcing the importance of carefully monitored human translation.

Additional genetic strategies, including porcine endogenous retrovirus inactivation, use of minipig backgrounds, and growth hormone receptor knockout to control post‑transplant organ growth, were acknowledged as important adjuncts to immunologic management. The group emphasised that immunosuppressive requirements must be considered in conjunction with the genetic profile of the donor animal rather than in isolation. A principal aim of donor genetic engineering is to reduce the burden of systemic immunosuppression, and nearly all modifications in current clinical‐grade pigs are directed at rejection and graft injury: carbohydrate‐antigen knockouts (GGTA1, CMAH, B4GALNT2), human complement regulators (CD46, CD55), coagulation regulators (THBD, EPCR), and innate or anti‐inflammatory transgenes (CD47, HMOX1, A20). Growth hormone receptor knockout and PERV inactivation are the principal non‐immunological exceptions. These additional edits are not trivial and carry their own complexity, therefore, finding the right balance between genetic modification and pharmacologic immunosuppression will be critical. Investigational strategies aimed specifically at further lowering immunosuppression include SLA (MHC) class I knockout with class II suppression (CIITA), HLA‐E or HLA‐G expression to temper NK‐cell responses, and transgenic costimulation blockade or checkpoint ligands (for example CTLA4‐Ig or PD‐L1). For transgenes in particular, the differential expression across tissues and functional units within an organ, as well as their persistence over time, will need to be thoroughly scrutinised.

### 4.4 Tolerance‑Oriented Strategies

The group identified the reduction and eventual replacement of lifelong systemic immunosuppression as a critical long‑term goal. Approaches including mixed hematopoietic chimerism, thymic transplantation, and combined organ‑thymic grafts have shown promise in preclinical settings, achieving prolonged survival and partial immune accommodation [45]. Durable tolerance has not yet been reliably demonstrated in large‑animal models, and ethical and logistical constraints currently limit broad clinical application. Importantly, thymokidney grafts derived from GGTA1 knockout pigs with no additional genetic modifications have been reliably accepted in baboon recipients for more than six months, with demonstrated donor‐specific hyporesponsiveness. Belatacept monotherapy ultimately led to rejection, indicating that durable drug‐free tolerance remains an unmet goal even in this favourable model. Mixed hematopoietic chimerism approaches are promising in principle but not yet considered ready for broad clinical application given persistent barriers to achieving sufficient durable engraftment in large‐animal models.

Nonetheless, specific platform elements, such as co‑transplantation of donor‑derived thymic tissue with kidney xenografts, have advanced to investigational use and may represent incremental steps toward tolerance induction despite short‐term success to date [46]. In parallel, encapsulation technologies and immune‑modulating scaffolds for cellular xenografts may offer practical strategies to reduce systemic drug exposure while maintaining graft protection.

### 4.5 Localised and Transgene‑Mediated Immunomodulation

Recent donor engineering has enabled graft‑intrinsic expression of immunomodulatory molecules, including ligands that inhibit T‑cell activation or enhance immune checkpoint signalling. These approaches have been applied for xenoislet transplants with significant success in NHP models [47]. These approaches aim to localise immunologic protection of the graft and reduce the systemic burden of immunosuppressive therapy. Complementary delivery platforms, such as drug‑eluting biomaterials, encapsulated cell devices, and perfusion‑based vascular coatings have demonstrated encouraging preclinical signals. The group concurred that these strategies warrant structured evaluation in combination with reduced‑intensity systemic immunosuppressive regimen.

### 4.6 Patient Monitoring and Management

Effective patient management in xenotransplantation extends beyond conventional transplant care to include advanced immunologic, microbiological, and molecular monitoring [48]. Donor‑derived cell‑free DNA, cytokine and chemokine profiling, and complement activity assays provide early insight into graft injury and immune activation. Protocol biopsies combined with biomarker surveillance have enabled timely intervention for rejection episodes in early clinical experiences and provide a means to enhance graft survival especially in combination with newly developing technologies (see also group seven). A comprehensive core monitoring framework should additionally include: coagulation and endotheliopathy markers (platelets, fibrinogen, D‐dimer, von Willebrand factor, syndecan‐1) to detect early vascular injury; molecular pathology and spatial transcriptomics for characterisation of xeno‐specific kidney pathology including glomerulopathy and thrombotic microangiopathy; and real‐time DSMB‐visible dashboards integrating laboratory, biomarker, imaging, and safety data with automated alerting and biobanking of serum/PBMC/plasma at baseline and key milestones. Critically, monitoring requirements must not systematically exclude participants lacking logistical or geographic access; trial budgets should include provision for travel support and validated remote sampling kits where available.

Patient selection remains a critical determinant of outcome. The group noted that candidates with limited allotransplant options, such as highly sensitised patients lacking preformed anti‑pig antibodies, may be appropriate for early trials, provided robust contingency strategies exist. Early experiences caution against compounding multiple high‑risk clinical factors in first‑in‑human studies and support prioritisation of physiologic stability and adherence capacity. Importantly, early clinical experience has highlighted the danger of compounding multiple extreme risk factors in first‐in‐human trials. A case combining advanced heart failure, concurrent LVAD implantation, and a combined thymokidney xenograft in a haemodynamically unstable recipient resulted in kidney xenograft loss, underscoring the need to avoid such compound high‐risk scenarios in early trials and to prioritise candidates with relative physiologic stability.

### 4.7 Summary Recommendations

The expert panel recommends that IXA endorse a tiered, adaptive approach to immunosuppression and patient management that:

- Aligns systemic immunosuppressive regimen with organ type and donor genetic background.
- Supports continued evaluation of targeted and graft‑intrinsic immunomodulatory strategies.
- Encourages incremental exploration of tolerance‑oriented pathways.
- Embeds advanced biomarker‑guided monitoring into clinical protocols and patient care.
- Promotes harmonisation across clinical trials while preserving flexibility for scientific innovation.

## 5 Working Group 4: Infectious Disease Risk and Surveillance

The group's deliberations reaffirmed that the prevention, detection, and management of infectious disease risk remain foundational to the safe clinical translation of xenotransplantation. It was also noted that xenotransplantation carries a potential risk of transmission of microorganisms from the donor to the immunosuppressed recipient [49]. In addition to infections associated with allotransplantation, xenotransplants carry the added possibility of exposures to organisms carried by the source animal [50]. While early clinical experience using carefully screened swine has not identified evidence of zoonotic transmission to recipients or close contacts, these risks remain central to public trust, ethical justification, and regulatory oversight.

The group recognised that substantial progress has been achieved since the initial international consultations on xenotransplantation. As discussed by all groups, advances in biosecure breeding, pathogen surveillance, donor selection, immunosuppression and molecular diagnostics have significantly reduced infectious risk and strengthened the scientific basis for cautious clinical application. Significant progress has been made regarding the microbiological risks associated with pig‐to‐human xenotransplantation since the original consultations [12, 14, 51, 52, 53, 54]. Some guidelines have been developed to govern clinical trials [55, 56, 57, 58]. Importantly, infection risk mitigation was viewed not as an adjunct to trial design, but as a core scientific and ethical requirement integrated across donor management, recipient surveillance, occupational health, and public health interfaces. This document will summarize current knowledge and future directions related to xenozoonosis.

### 5.1 Lessons from Preclinical and Early Clinical Experience

The group reviewed cumulative data from large‑animal models and early human experiences, noting that infectious complications influencing graft performance have most often reflected donor‑derived porcine viruses rather than true cross‑species infection. It was noted that some common zoonotic porcine viruses are known, including Hepatitis E virus genotype 3 (HEV3) and swine influenza [49, 59, 60]. Other viruses such as porcine cytomegalovirus/porcine roseolovirus (PCMV/PRV) have emerged as a critical determinant of xenograft outcomes. Although not zoonotic in the traditional sense in that it does not infect human cells, it may cause systemic manifestations when introduced into the recipient via xenotransplantation [61, 62]. PCMV/PRV has been shown to induce systemic inflammation, coagulopathy, and accelerated graft failure in non‑human primate models and has likely contributed to reduced graft survival in at least one human cardiac xenotransplant [62, 63]. These findings were considered decisive evidence that exclusion of PCMV/PRV from donor herds is essential for clinical trials.

Other porcine viruses of relevance include porcine circoviruses which are ubiquitous in swine populations and have not been conclusively associated with human disease; however, the demonstration of PCV3 replication in porcine cardiac xenograft baboon recipients [64] and recent reports of human circovirus disease in transplant settings suggest that circoviruses warrant surveillance and, where feasible, excluded in pigs bred as organ donors [65]. Porcine lymphotropic herpesviruses, while associated with lymphoproliferative disease in immunosuppressed swine, have not been shown to infect human or non‑human primate cells in vitro, but remain part of recommended donor screening panels.

### 5.2 Porcine Endogenous Retroviruses

Porcine endogenous retroviruses (PERVs) were extensively discussed given their historical significance and public health implications. The group reaffirmed that PERV‑A and PERV‑B are present in all pigs, while PERV‑C is absent in some strains. Recombinants between PERV‐A and PERV‐C, called PERV‐A/C, are found in the circulation and in the genome of somatic pig cells, indicating ongoing recombination activity of PERVs in vivo [66].

Human cells have receptors for PERV‐A, ‐B and A/C, but not for PERV‐C. In vitro, PERV infects selected, generally transformed, human cell lines [67, 68, 69]. Infection of primary human cells can be achieved for some primary cell lines using high titers of virus or contact with highly expressing pig cells. PERV has never been transmitted in preclinical or clinical trials of pig tissues [70].

The group emphasised that interpretation of post‑transplant PERV surveillance requires careful consideration of microchimerism, particularly the presence of circulating porcine cells or DNA, which can give rise to false‑positive results. Accordingly, PERV testing should be performed in accredited laboratories, with confirmatory analysis conducted by designated reference facilities. This approach was viewed as essential to prevent unwarranted antiviral therapy, unnecessary patient isolation, or inappropriate regulatory responses.

The group agreed with Working Group 1 that the risk mitigation strategies for PERV, should start with the selection of donor pigs lacking PERV‑C, breeding for low‑expression PERV‑A/B profiles, antiretroviral susceptibility, and/or CRISPR/Cas‑based genomic inactivation of PERVs, were recognised as important advances that further support cautious clinical translation. The group noted that available antiretroviral agents demonstrate in vitro activity against PERV and could be considered as part of contingency planning rather than routine prophylaxis.

### 5.3 Donor Surveillance, Biosecure Breeding, and Trial Design

The group agreed with Working Group 1 that effective infectious risk mitigation depends on the maintenance of biosecure breeding facilities producing designated pathogen‑free donor pigs. The underlying principle is that microorganisms excluded from the donor herd or individual animals should not be transmitted to recipients. This requires clearly defined exclusion lists, regular microbiological surveillance, and validated assays tailored to pathogens of relevance in immunocompromised humans. Thus, an “exclusion list” is required for each colony to create a “designated pathogen free” (DPF) donor for those regions endemic pig diseases. Local regulatory guidelines for breeding and for clinical trials must consider for such lists a group of microorganisms likely to be important for immunocompromised recipients [53, 59, 71].

To achieve this goal, routine microbiological testing of animals must be conducted for animals raised in a biosecure environment to prevent introduction of adventitious agents [49, 53]. This requires protocolised surveillance strategies and sensitive and specific PCR‐based and immunological (serologic) assays for organisms considered to be potential human pathogens [72, 73].

Routine testing strategies should integrate molecular and serologic methods and be supported by protocolised surveillance schedules as part of clinical trial design. As discussed in consultation with groups three and seven there are emerging diagnostic approaches, including metagenomic and both targeted and unbiased next‑generation sequencing, viewed as valuable adjuncts for detecting unknown or unexpected pathogens [53], particularly in the context of longitudinal recipient monitoring. However, the group acknowledged persistent gaps in assay validation for certain porcine pathogens and limited understanding of their biological behaviour in human hosts, emphasising the need for continued methodological development.

Approaches such as early weaning, Caesarean delivery, and embryo transfer [74] were recognised as effective tools for reducing vertical transmission of selected porcine viruses and should be incorporated into breeding strategies where appropriate.

### 5.4 Infection Control, Occupational Health, and Public Health Considerations

Beyond donor and recipient surveillance, the group stressed the importance of infection control and occupational health planning at hospital centres undertaking clinical xenotransplantation [57]. Existing evidence supports the adequacy of standard universal precautions for most clinical scenarios, with additional measures tailored to the microbiological profile of donor herds and study‑specific risk assessments.

Occupational health protocols should address potential exposure of healthcare and laboratory personnel to blood and body fluids from donor pigs or xenograft recipients, including management of needlestick injuries or mucosal exposures. While post‑exposure antiviral therapy could be considered in exceptional circumstances involving donors with active PERV replication, the group noted that no evidence of PERV infection has been observed in individuals with extensive occupational exposure to pigs, such as piggery staff, abattoir workers, butchers or meat handlers.

The group supported biobanking of baseline sera and blood cells from xenograft recipients to facilitate future epidemiological investigation should infectious syndromes arise. Further testing for swine pathogens in the close contacts of xenotransplant recipients and hospital staff might be reserved for infectious symptoms or demonstrated infection in the recipient.

In summary, with validated, specific, and sensitive detection methods and testing strategies for monitoring of source animals and recipients, the safety of xenotransplantation can be advanced until further data are available from clinical trials.

### 5.5 Summary Recommendations

- Infectious disease risk mitigation remains a core scientific, ethical, and regulatory requirement for clinical xenotransplantation.
- Donor‑derived porcine viruses, particularly PCMV/PRV, have demonstrated clear relevance to graft outcomes and must be rigorously excluded.
- No evidence of human infection with porcine endogenous retroviruses (PERV) has been documented, but structured surveillance and validated testing remain essential.
- Biosecure breeding, designated pathogen‑free herds, and validated microbiological assays must be integrated into trial design.
- Infection control and occupational health strategies should be proportionate, evidence‑based, and aligned with donor herd epidemiology.
- Continued large‑animal studies, methodological innovation, and public health transparency are required to responsibly advance clinical trials.

## 6 Working Group 5: Ethical and Legal Frameworks

The working group acknowledged that ethical, legal, and societal considerations surrounding xenotransplantation have become increasingly considered since the 2018 Changsha Communiqué [14], reflecting the transition of the field from preclinical experimentation to early clinical application. First‑in‑human experiences with heart and kidney xenotransplantation, use of expanded‑access pathways, and the initiation of regulated clinical trials have shifted ethical analysis from largely theoretical deliberation toward practical governance challenges arising in real clinical contexts [37]. These developments underscore the need to refine guidance relating to ethical and legal frameworks in particular in recipient selection, informed consent, oversight mechanisms, public engagement, and equitable access, while maintaining consistency with the foundational principles articulated in the original Changsha Communiqué [11].

### 6.1 Recipient Selection

The working group agreed with the other Working Groups, specifically Working Group 3, that recipient selection is an area in which ethical thinking has evolved most substantially since 2018. The Changsha Communiqué recommended that trial participants be limited to individuals for whom no adequately effective alternative therapy was available. While this principle remains central, the group recognised that the varied realities of international clinical transplantation systems, including prolonged waiting times, organ scarcity, and differential access to allotransplantation in line with equitable access as outlined by the Santander statement [75].

In particular, the group noted increasing consensus that patients who are technically eligible for allotransplantation but face a high likelihood of death before organ availability may constitute an ethically appropriate population for early xenotransplantation trials. This includes individuals with extreme sensitisation, prolonged predicted waiting times, limited vascular access, or poor suitability for durable mechanical support. In such cases, xenotransplantation may represent a proportionate intervention aligned with the principles of beneficence and justice, even when formal allotransplant eligibility exists. Early clinical kidney xenotransplantation experiences at Massachusetts General Hospital in 2024 and 2025 were cited as illustrative of this broadened ethical framing [29, 76].

Accordingly, the group supported updating recipient selection guidance to include patients for whom there is no effective alternative therapy available and/or for whom death before transplant would be likely, if participants demonstrate a clear understanding of risks and a capacity to comply with lifelong follow‑up requirements.

### 6.2 Informed Consent

The group reaffirmed informed consent as a foundational ethical requirement for xenotransplantation clinical trials, while emphasising that consent processes must evolve to reflect the complexity and uncertainty inherent in first‑in‑human and early‑phase studies. The 2018 update of the Changsha Communiqué emphasised consent from motivated patients willing to accept stringent conditions, alongside education of patients and close contacts to promote compliance and minimise risks to individuals and society [77]. The working group endorsed retention of this framework, with refinement to emphasise therapeutic adherence, optimisation of clinical outcomes, and explicit education regarding infectious disease risks.

The group noted that xenotransplantation consent processes often require prolonged and iterative engagement, particularly in non‑emergent settings. Tiered consent models, including consent to be approached, consent for evaluation and education, and consent for the procedure itself, were regarded as potentially valuable mechanisms for supporting patient understanding and voluntariness. The use of independent educators or advocates, where feasible, was viewed as a constructive approach to mitigating conflicts of interest and reinforcing patient‑centred decision‑making.

Special ethical challenges arise in paediatric xenotransplantation, particularly regarding surrogate consent and the potential role of adolescent assent. These challenges closely parallel issues encountered in paediatric/adolescent allotransplantation but remain under‑examined in the xenotransplantation context [78, 79, 80]. The group encouraged careful ethical scrutiny and experience‑sharing as consideration of paediatric applications emerges.

### 6.3 Close Contacts and Public Health Responsibilities

The working group devoted substantial discussion to the ethical status of close contacts, including caregivers, household members, and sexual partners [81]. While cooperation of close contacts in infection surveillance was recognised as highly desirable from a public health perspective, the group concluded that conditioning a patient's participation on formal consent from bystanders raises significant ethical concerns [82]. Such requirements risk undermining respect for personal autonomy by transferring decisional authority from a decision capable individual to third parties.

Instead, the group supported an education‑focused approach in which close contacts are provided with clear information regarding potential infectious risks, symptoms warranting evaluation, and recommended monitoring practices. While some jurisdictions may mandate cooperation in surveillance activities, the panel agreed that requiring bystander consent as a precondition for enrolment cannot be ethically justified in most circumstances and will rely upon the various jurisdictions to decide this.

### 6.4 Legislative and Regulatory Considerations

The working group observed that, despite rapid scientific and clinical progress, relatively few jurisdictions have undertaken substantive revision of xenotransplantation‑specific legislation since 2018. Regulatory approaches remain heterogeneous, reflecting differences in national legal traditions and health‑system structures. The IXA Council has consistently provided guidance documents including the most recent series of “White Papers” [37, 38, 83, 84] and the IXA Ethics Committee as a source of expert guidance for investigators and regulators; however, legislative authority remains the prerogative of national governments.

As per the IXA's prior recommendations, the group recommended that jurisdictions pursuing xenotransplantation establish mechanisms for regular review and updating of relevant regulations and laws. Periodic reassessment was viewed as an important safeguard to ensure that governance frameworks remain aligned with scientific advances, evolving ethical norms, and public expectations. Switzerland's practice of revisiting xenotransplantation regulations at regular intervals was cited as a potential example of best practice [85].

### 6.5 Public Engagement

Public engagement was reaffirmed as a core component of responsible xenotransplantation governance, consistent with the Communiqué’s emphasis on transparency and public involvement. The working group recognised that meaningful public engagement extends beyond formal regulation and includes education, dialogue, and responsiveness to societal concerns and the importance of engagement of the public in clinical xenotransplantation [86].

Practical strategies discussed included the inclusion of patient and community representatives on institutional and national oversight bodies, targeted education of healthcare professionals, proactive and responsible engagement with media following xenotransplantation procedures, and development of accessible educational materials and public forums. The group also highlighted the great importance of media literacy initiatives aimed at reducing misinformation and sensationalism surrounding xenotransplantation.

The inclusion of patient perspectives in public‑facing communications was viewed as a potentially powerful means of contextualising xenotransplantation in terms of lived experience and unmet medical need. Such engagement should be voluntary, ethically guided, and sensitive to the diverse interests of patients, families, and communities [87].

### 6.6 Oversight Mechanisms and Patient Advocacy

The working group emphasised the importance of robust oversight to maintain scientific integrity and public trust. Institutional review boards, national regulators, and independent ethics committees were identified as central components of this framework. The IXA Ethics Committee was reaffirmed as a standing body capable of providing expert, confidential advice to centres pursuing xenotransplantation and, where appropriate, facilitating access to patient advocates. Additionally, patient advocacy has been argued for in the literature as a powerful means to overcome trust issues [88].

Consistent with emerging ethical literature, the group encouraged xenotransplantation centres to incorporate patient advisory structures into study design and conduct. Meaningful patient involvement was viewed as a mechanism to foster patient‑centred research culture, clarify participant priorities, support retention, and enhance transparency [89].

### 6.7 Equitable Access

The working group revisited the issue of equity. The Changsha Communiqué called upon WHO to promote equitable access to successful xenotransplantation products. However, the group agreed that equity considerations arise well before regulatory approval [90]. Revising this principle to encompass equitable access to xenotransplantation clinical trials was viewed as ethically important, particularly as early clinical opportunities may be limited and highly selective. In line with the mission of IXA and in line with the Santander Statement [75] ensuring that access pathways do not systematically disadvantage vulnerable or marginalised populations remains a critical ethical challenge as the field continues to advance.

### 6.8 Summary Recommendations

The expert panel recommended that IXA update and refine its ethical and governance guidance for clinical xenotransplantation in a manner that:

- Recognised an expanded ethical framework for recipient selection that includes patients with no effective or timely alternative therapy and those at high risk of death while awaiting allotransplantation;
- Endorsed robust, iterative, and where feasible tiered informed‑consent processes that emphasise patient understanding, voluntariness, long‑term compliance, and explicit education regarding infectious and societal risks;
- Supports education‑based engagement of close contacts while rejecting bystander consent as a precondition for enrolment, except where explicitly mandated by law;
- Encouraged regular national review and updating of xenotransplantation‑related regulations and legislation, informed by scientific progress and evolving ethical norms;
- Promotes transparent and proactive public engagement, including responsible media interaction, patient and community representation in oversight structures, and accessible educational initiatives;
- Affirms the role of independent ethics oversight and patient advocacy, including the availability of the IXA Ethics Committee as a confidential advisory resource and the inclusion of patient advisory boards in study design and conduct;
- Emphasised that considerations of equity apply throughout the translational pathway, including fair access to xenotransplantation clinical trials as well as approved clinical applications.

## 7 Working Group 6: International Collaboration and Governance

The working group recognised that international collaboration and governance are increasingly central to the responsible clinical translation of xenotransplantation [14]. Advances in genetic engineering of donor pigs, together with the initiation of early‑phase clinical trials, have transformed xenotransplantation from a largely theoretical regulatory challenge into a practical, multinational enterprise. These developments underscore the need for updated international coordination mechanisms, overarching guidance documents, harmonised regulatory approaches, and shared governance structures capable of supporting safe, ethical, and scientifically rigorous clinical implementation.

The Changsha Communiqué articulated several overarching principles to guide WHO positions on regulatory requirements for xenotransplantation clinical trials. As xenotransplantation approaches broader clinical application, the group considered it both timely and necessary to revisit the scope and operationalisation of these principles. Updates to the 2018 recommendations, identified through subsequent WHO consultations and reflected in comparative tables within this report, illustrate the emergence of new governance needs driven by technological progress and clinical readiness.

### 7.1 Global Information Sharing and Registries

The working group reaffirmed the longstanding importance of comprehensive global information sharing in xenotransplantation which aligned with international consensus in transplantation [75]. Established originally in consultation with the WHO, the IXA has supported the maintenance of a Global Inventory to document xenotransplantation activities worldwide [83, 91], recognising that transparent access to high‑quality data is fundamental to public trust, scientific progress, and regulatory oversight. As clinical activity increases, the limitations of fragmented or nationally siloed reporting systems have become increasingly apparent.

The group supported the expansion of an international xenotransplantation inventory, expanding it to a more comprehensive registry that is independent of individual clinical trial platforms and capable of capturing longitudinal data across jurisdictions. At this stage, the working group endorsed IXA's recommendation for the creation of a WHO/IXA‑led xenotransplantation trial registry, incorporating the principles outlined in the IXA White Paper [83]. Expansion of existing platforms or integration with established international infrastructures, such as the Global Observatory on Donation and Transplantation (https://www.transplant‐observatory.org), was viewed as a pragmatic and potentially efficient pathway to achieving this goal.

### 7.2 International Harmonisation of Regulatory Processes

The working group revisited IXA's earlier proposal for international harmonisation of regulatory processes governing xenotransplantation. As clinical trials increasingly involve multinational collaboration, the absence of aligned application pathways risks inefficiency, duplication of effort, and inconsistent ethical and safety standards. The group supported continued exploration of a harmonised application framework that integrates international best practices as outlined by the WHO‐IXA meetings including the 2018 update [77], while respecting national regulatory sovereignty.

Key elements of such harmonisation include alignment in donor animal breeding and facility standards, core trial design requirements, biosecurity measures, and minimum data‑reporting expectations. A unified application pathway is outlined in accompanying Table 1. This proposes a unified application process for xenotransplantation trials, that was viewed as both feasible and desirable, particularly for early‑phase trials seeking parallel approvals in multiple jurisdictions. The group emphasised that harmonisation need not imply uniformity in every detail, but rather convergence on core principles of safety, ethical integrity, and scientific rigor.

**TABLE 1:** Proposed unified application pathway for xenotransplantation clinical trials.

Review component	Responsible body	Scope and function	Framework/examples
Scientific review	Centralised international expert panel	Independent evaluation of scientific validity, preclinical evidence, trial design, and risk–benefit profile; promotes consistency across jurisdictions	Modelled on an international xenotransplantation expert body (e.g., IXA Ethics/Scientific Committee framework)
Ethical review	Local or regional Institutional Review Boards (IRBs) / Ethics Committees	Assessment of ethical acceptability, participant protection, informed consent, and societal considerations within the local context	Harmonised ethical framework (e.g., ICH GCP; WHO Changsha Communiqué)
Regulatory review	National regulatory authorities	Evaluation of compliance with national laws and regulatory requirements, including safety, manufacturing standards, and clinical trial authorisation	Agencies such as FDA (USA), EMA (EU), TGA (Australia), and equivalent bodies
Ministerial/central authority approval	National government or designated central authority (where applicable)	Final authorisation for high‐risk or novel procedures, reflecting broader public health, policy, and societal considerations	Required in selected jurisdictions (e.g., New Zealand, China)

### 7.3 Governance Models and Oversight Structures

With respect to governance, the working group recognised the potential value of tiered oversight structures incorporating regional nodes, operating within a globally coordinated framework. Such an approach could support alignment with internationally agreed principles while allowing appropriate responsiveness to regional and national regulatory, ethical, and sociocultural contexts. Regional nodes, linked through IXA and WHO coordination mechanisms, could facilitate structured information sharing, capacity building, and peer‑to‑peer regulatory learning, thereby strengthening consistency and transparency across jurisdictions. This discussion occurred in the context of several jurisdictions reporting active review or reconsideration of their xenotransplantation regulatory frameworks, including Europe [92], South Korea [93], Australia [94], New Zealand and the United States [95], highlighting the importance of governance models that are both harmonised and adaptable as the field progresses toward broader clinical translation.

The group highlighted that effective governance in xenotransplantation requires sustained interaction between scientific, regulatory, and ethical expertise. In this context, a continuously updated, internationally accessible xenotransplantation platform maintained by IXA encompassing scientific, regulatory, and ethical dimensions was viewed as a potentially valuable tool for both investigators and regulators.

### 7.4 Funding, Capacity Building, and Inclusion of LMICs

The working group placed particular emphasis on mechanisms for capacity building, especially with respect to low‑ and middle‑income countries (LMICs). Enabling LMICs to participate meaningfully in xenotransplantation research and, where appropriate, clinical implementation was viewed as an equity issue and a governance imperative. Building regulated clinical capacity in these settings may also serve as an important strategy to reduce the risks of organ trafficking and unregulated xenotransplantation practices.

The group encouraged inclusion of dedicated mechanisms to support LMIC engagement, including regulatory training, infrastructure development, and collaborative clinical research partnerships. Well‐regulated xenotransplantation jurisdictions, including the United States, several European countries, Australia, and New Zealand, were identified as potential sources of regulatory mentorship and scientific collaboration. Core regulatory themes shared across jurisdictions, as summarised in Table 2, can serve as a foundation to identify and build cooperative capacity‑building efforts for international collaboration and governance.

**TABLE 2:** Provides an overview of select regulatory frameworks and approval pathways across jurisdictions that are evaluating undertaking clinical xenotransplantation. The table provides an overview from some regulated xenotransplantation jurisdictions, including the United States, Europe, Australia, and New Zealand. These provide a foundation for regulatory mentorship and scientific collaboration. The shared regulatory themes outlined in Table 2 are provided to help frame and inform cooperative capacity‐building efforts and support the development of improved harmonised international governance frameworks for xenotransplantation.

National jurisdiction	Key agencies involved	Approval layers/process	Unique or required features
Argentina	ANMAT; INCUCAI; CONABIA; IEC	Ethical, biosafety, transplant policy and regulatory oversight	GM pig oversight; transplant compatibility frameworks
Australia	TGA; HREC; OGTR	Ethical, regulatory and biosafety approval	CTA scheme; OGTR oversight of genetically modified source animals
China	NMPA; NHC; IRBs	Centralised approval, ethics and biosafety review	Class III classification; CPC approval under xenotransplantation guidelines
European Union (EU)	EMA; National Authorities; Ethics Committees	CTIS submission and coordinated regulatory review	ATMP classification; zoonotic risk assessment; CTIS platform
India	CDSCO; ICMR; DBT	NDCTR and coordinated multi‐agency approval	Integrated multi‐agency oversight requirement
Japan	MHLW; PMDA; Ethics Committees	Regenerative medicine and clinical trial regulatory pathways	Integration within advanced therapy frameworks; alignment with international standards
Mexico	COFEPRIS; CENATRA	Licensing, documentation and registry reporting	Transplant‐aligned framework with limited xeno‐specific regulation
New Zealand	Medsafe; GTAC; HDEC; Minister of Health	Scientific, ethical, regulatory and ministerial approval	DPF pigs; PERV monitoring; ministerial authorisation
Philippines	FDA; CDRR; PHREB	FDA licensing and IRB/ERB approval	Source animal selection; zoonotic risk mitigation; genetic modification identification; long‐term surveillance requirements
Republic of Korea (South Korea)	MFDS; Ministry of Health & Welfare; IRBs	ARMBA‐based multi‐agency ethical, regulatory and clinical trial approval	Advanced Regenerative Medicine and Biopharmacology Act; coordinated national oversight aligned with international guidance
Spain	AEMPS; CEIm	Ethical review, IMPD submission and REec registration	Transparency via REec; alignment with EMA regulatory processes
United Kingdom	MHRA; HRA; Ethics Committees	CTA submission, ethical review and safety data evaluation	Detailed safety requirements including donor screening, genetic modification controls and infection surveillance
United States	FDA; PHS	IND submission with long‐term post‐transplant surveillance	Strong emphasis on zoonotic risk, informed consent and public health monitoring

### 7.5 Role of WHO and Ongoing Global Coordination

The working group reaffirmed the central role of WHO in promoting global awareness of both the potential benefits of xenotransplantation and the risks associated with unregulated practice. Recommendations made in 2018 emphasising WHO's public‑facing leadership were considered to remain highly relevant and have specifically informed deliberations at the accompanying 2025 WHO Global Consultation Meeting. IXA recommended that WHO should continue to play a significant place in promoting public awareness of both the benefits as well as the possible dangers associated with unregulated xenotransplantation.

In concluding discussions, the group noted that the 2018 WHO Global Consultation Meeting had been successful in establishing a shared ethical and regulatory foundation, now being refined through updated guidance aligned with contemporary clinical and scientific realities. Participants at the 2025 consultation consistently emphasised the continued need for close and sustained interaction at the global level among scientific innovators, regulators, ethicists, and implementing clinicians.

Within this context, the working group concluded that a unified or harmonised application process for xenotransplantation trials is both feasible and increasingly necessary. By integrating regulatory diversity with rigorous international oversight and guidance through IXA, TTS and WHO collaboration, it may be possible to accelerate responsible innovation while safeguarding public health, ethical standards, and societal trust.

### 7.6 Summary Recommendations

The expert panel recommends that IXA and WHO continue to strengthen international collaboration and governance in xenotransplantation in a manner that:

- Supports the development of a WHO and IXA‑led international xenotransplantation registry that is independent of individual trial platforms and integrated where feasible with established global transplant registries. It should build on the current WHO/IXA inventory;
- Encourages progressive international harmonisation of regulatory processes, including aligned core requirements for donor standards, facility oversight, trial design, and data reporting, while respecting national regulatory authority;
- Promotes tiered governance models with regional nodes to facilitate coordination, peer learning, and responsiveness to local regulatory contexts;
- Prioritises funding and capacity‑building mechanisms to enable meaningful participation by low‑ and middle‑income countries, thereby supporting equity and reducing risks of unregulated xenotransplantation;
- Reinforces the leadership role of WHO in public awareness, global coordination, and ethical oversight of xenotransplantation;
- Endorses the maintenance of a continuously updated, internationally accessible xenotransplantation platform coordinated by IXA to support scientific, regulatory, and ethical best practices.

## 8 Working Group 7: New Technologies for Xenotransplantation

The working group provided a substantial case that the scope of “new technologies” relevant to xenotransplantation has expanded substantially since the 2018 Communiqué revision. At that time, technological innovation was largely focused on gene‑edited donor pigs and emerging targeted immune modulation. Both remain central to xenotransplantation. The scope of innovation has expanded to include digital and molecular technologies. Artificial intelligence and predictive analytics are being explored for donor and recipient matching and rejection risk modelling. Multi‐omics platforms provide deeper insights into graft compatibility and immune dynamics. Novel biomarkers such as donor‐derived cell‐free DNA, cytokines, and complement activity markers offer promise for early detection of rejection or infection. Real‐time graft monitoring tools and the integration of these technologies into clinical trial workflows represent important steps toward safer and more effective clinical translation. Clinical trials should incorporate the broader landscape of new technologies. IXA should play an active role in promoting their responsible development, standardisation, and clinical validation.

### 8.1 Donor and Recipient Selection and AI‑Enhanced Allocation

The working group highlighted the growing role of computational methods in improving donor and recipient selection. Advances in artificial intelligence and bioinformatics now enable integration of whole‑genome sequencing, pathogen screening, and genetic integrity assessment of donor animals into unified risk stratification frameworks. AI‑supported analyses can assist in prioritising donor pigs based on infectious risk, transgene composition, and suitability for specific clinical protocols [96, 97], including decisions regarding porcine endogenous retrovirus inactivation strategies. These approaches support the donor genetic strategies and immunosuppressive tailoring discussed in the other Working Groups, ensuring that immunologic risk, donor design, and systemic immunosuppression are considered as interdependent variables rather than in isolation.

On the recipient side, machine‑learning approaches have the potential to identify patients least likely to receive timely allotransplantation and therefore most likely to benefit from xenotransplantation [98], while avoiding compounding multiple high‑risk clinical factors, reinforcing the patient‑selection principles outlined by the immunosuppression and patient‑management groups.

The group emphasised the need for standardisation, including harmonised reference datasets, validated analytic pipelines, harmonized reporting templates, and inter‑centre proficiency testing, possibly coordinated or endorsed by IXA, to ensure comparability and transparency across programs [99].

The group also reviewed current approaches for xenoantibody detection and pre‑transplant screening. Existing assays, including flow cytometric and complement‑dependent cytotoxicity crossmatches, provide valuable information but have recognised limitations related to cell source, transgene expression, and sensitivity as seen in current cases of xenotransplantation [31, 100]. The group supported continued development of donor‑specific assays and bioinformatics‑enabled antibody profiling tools to improve precision in pre‑transplant risk assessment.

### 8.2 Non‑Invasive Monitoring Tools and Biomarkers

The working group identified non‑invasive monitoring as a critical technological enabler that directly complements the biomarker‑guided patient‑management strategies suggested in combination with Working Groups 2 and 3. Donor‑derived cell‑free DNA has been validated in allotransplantation [101] and is being applied in cardiac and renal xenotransplantation [102, 103], demonstrating greater sensitivity than conventional functional markers when used in a complementary manner. The availability of assays capable of detecting porcine cell‑free DNA creates a realistic pathway toward routine “liquid biopsy” approaches in xenotransplantation.

Multiplex immune‑profiling technologies allow broad assessment of cytokine and chemokine responses from small sample volumes and are particularly valuable in nonhuman primate studies and paediatric contexts. The group agreed that biomarkers should now transition from exploratory investigation toward systematic incorporation into preclinical and clinical protocols. Standardisation of assays, thresholds, and reporting across centres was viewed as an important priority for IXA engagement.

Building on these tools, the group discussed emerging concepts of virtual biopsy and xenotransplant‑specific risk scores, integrating biomarkers, clinical variables, and predictive analytics to enable individualised monitoring and early intervention. The development of integrated dashboards combining laboratory data, biomarker signals, imaging, and safety indicators was identified as a key enabler of AI‑supported clinical decision‑making, contingent on robust data standards, interoperability, and governance frameworks.

### 8.3 Tissue‑Based Precision Diagnostics

The working group reaffirmed that tissue‑based diagnostics remain indispensable for defining rejection phenotypes and guiding immunosuppressive management, as discussed extensively in Working Groups 2 and 3. Advanced multimodal biopsy analysis enables precise characterization of xenoimmune injury patterns, complement activation, and immune‑cell infiltration, thereby informing targeted therapeutic strategies. Maximising diagnostic yield from limited biopsy material was identified as a critical challenge, particularly in early clinical studies [97].

The group highlighted the need for a comprehensive pig‑to‑human diagnostic repository integrating histopathology, immune profiling, donor genetics, and clinical outcomes. Standardisation was considered essential, including consensus diagnostic criteria analogous to the Banff classification in allotransplantation, harmonised standard operating procedures, and protocols for data and metadata exchange. Such frameworks would support shared analyses, accelerate collective learning, and enable development of multimodal “digital twin” models integrating tissue, biomarker, and clinical data.

Advanced diagnostic techniques combining histopathology, immunohistochemistry, multiplex immunofluorescence, electron microscopy, and targeted molecular profiling from a single biopsy core were viewed as increasingly feasible and clinically relevant. When coupled with AI‑enabled digital pathology pipelines, these approaches allow detailed spatial and cellular characterisation of xenoimmune responses, complement activation, and graft injury. As outlined also by Group 1, both groups agreed that the importance of species‑specific probe design to overcome interspecies gene homology and improve diagnostic precision was a significant focus.

Integration of tissue‑based findings with non‑invasive biomarkers and clinical data into comprehensive xenograft precision reports was identified as an important step toward harmonised interpretation and personalised therapeutic decision‑making.

### 8.4 Integration of Multi‑Omics Approaches

The working group recognised multi‑omics technologies as transformative tools for future advances in immunosuppressive optimisation and tolerance‑oriented strategies. By linking transcriptomic, proteomic, and metabolic signatures with clinical phenotypes, these tools provide mechanistic insights that may guide donor pig engineering, identify actionable immune pathways, and inform development of next‑generation immunotherapies, directly supporting the long‑term objectives outlined by Working Group 3.

Emerging modalities such as spatial transcriptomics, single‑cell RNA sequencing, imaging mass spectrometry, and integrated complement profiling enable high‑resolution mapping of immune and parenchymal interactions within xenografts. The group acknowledged that these technologies remain resource‑intensive and are currently limited to specialised centres.

To support broader adoption, the group emphasised the need for structured capacity building, including training in data management and interpretation, systematic biobanking of tissues and biofluids, standardised sampling protocols, and shared reference datasets. AI and machine‑learning approaches were viewed as essential for integrating multi‑omic data with histology and clinical variables, enabling predictive modelling and identification of rejection signatures. While many applications remain research‑focused, these tools are already informing donor pig engineering and the development of targeted immunotherapeutic strategies.

### 8.5 Integrated and AI‑Driven Transplant Care

The working group concluded that integrated, AI‑driven clinical workflows represent a convergence point between new technologies and patient‑management strategies. Predictive models combining biomarkers, tissue diagnostics, and clinical variables may support real‑time adjustment of immunosuppression, early identification of rejection trajectories, and safer adaptive clinical trial designs. These approaches align directly with the tiered, biomarker‑guided management paradigm recommended by Working Group Three.

AI‑based allocation systems may improve donor–recipient matching and prioritisation, while adaptive trial designs incorporating early surrogate endpoints and real‑time monitoring could enhance both safety and efficiency in early‑phase studies. The group also highlighted the potential for patient‑centred digital tools, including carefully curated AI‑based educational platforms, to support informed decision‑making, adherence, and long‑term engagement, provided they are developed in alignment with IXA and professional society guidance.

### 8.6 Interface With Immunosuppression and Patient Management

The working groups jointly affirm that new technologies in xenotransplantation should not be viewed as adjunctive or exploratory, but as integral components of immunosuppressive strategy development, patient monitoring, and clinical decision‑making. Biomarker‑based surveillance, tissue‑level precision diagnostics, and AI‑enabled predictive models collectively provide the infrastructure required to move xenotransplantation beyond empiric regimen‑based care toward adaptive, personalised management. Close alignment between technological innovation and immunologic strategy is essential to maximise graft survival, minimise toxicity, and support ethical and safe clinical translation1.

### 8.7 Summary Recommendations

The expert panel recommends that IXA support the integration of new technologies into clinical xenotransplantation in a manner that:

- Recognises artificial intelligence, digital health tools, and advanced molecular diagnostics as core enablers of safe and effective clinical translation;
- Encourages standardisation and validation of AI‑supported donor and recipient selection tools, xenoantibody profiling methods, and pathogen‑screening pipelines;
- Promotes systematic incorporation of non‑invasive biomarkers, including donor‑derived cell‑free DNA and multiplex immune assays, into preclinical and clinical protocols;
- Supports the development of harmonised tissue‑based precision diagnostic frameworks tailored to pig‑to‑human transplantation and integrated with digital pathology and AI analytics;
- Facilitates responsible adoption of multi‑omics technologies through capacity building, shared repositories, standardised protocols, and data‑sharing frameworks;
- Endorses integrated, AI‑driven clinical workflows and adaptive trial designs that combine molecular, tissue‑based, and clinical data to enable personalised care and real‑time decision‑making.

## 9 Consolidated Summary Recommendations

### 9.1 Staged Clinical Translation and Scientific Justification

- IXA, in partnership with WHO, should reaffirm that clinical xenotransplantation proceeds only through staged, incremental introduction, grounded in robust preclinical evidence and justified by clearly articulated unmet clinical need, with early trials prioritising safety, feasibility, and biological insight over comparative efficacy.

### 9.2 Minimum Effective Donor Standards

- IXA should define, and WHO should endorse, minimum effective standards for genetically engineered porcine source animals, emphasising functional necessity, reproducibility, and biosafety rather than maximal genetic modification, with recognition of organ‑specific requirements.

### 9.3 Source Animal Biosecurity and Infectious Risk Mitigation

- WHO and IXA should continue to jointly mandate biosecure breeding of designated pathogen‑free source herds, with explicit exclusion of pathogens demonstrated to compromise graft outcomes (notably PCMV/PRV), validated surveillance for PERV, and transparent international reporting of infectious events.
- Long‑term recipient monitoring and proportionate public‑health surveillance should remain non‑negotiable conditions of clinical participation, consistent with Changsha principles.

### 9.4 Harmonised Clinical Trial Design and Oversight

- IXA should promulgate harmonised core requirements for early‑phase xenotransplantation trials, including patient selection criteria, predefined safety endpoints, adaptive stopping rules, and independent data safety monitoring.
- WHO should encourage regulatory convergence across jurisdictions, while respecting national authority, to reduce duplication of risk and promote consistent ethical and scientific standards.

### 9.5 Patient Selection and Ethical Eligibility

- IXA and WHO should endorse an updated ethical framework for recipient selection, extending beyond a strict “last‑resort” paradigm to include patients with a high likelihood of death before allotransplantation or with insurmountable barriers to timely access, provided informed consent and long‑term compliance are demonstrable.

### 9.6 Informed Consent and Long‑Term Obligations

- IXA should recommend, and WHO should support, robust, iterative informed‑consent processes that explicitly address uncertainty, infectious risk, long‑term surveillance obligations, and societal considerations, with encouragement of tiered consent and independent educational support.

### 9.7 Immunosuppression and Patient Management

- IXA should promote a tiered, adaptive approach to immunosuppression, aligned with organ type and donor genetics, incorporating costimulation blockade, targeted innate immune modulation, and biomarker‑guided management rather than fixed empiric regimens.
- Tolerance‑oriented strategies should be encouraged as long‑term objectives, progressing cautiously through structured preclinical and investigational clinical evaluation.

### 9.8 Integration of New Technologies

- IXA, with WHO endorsement, should recognise advanced diagnostics, biomarkers, digital health tools, and artificial intelligence as core enablers of responsible clinical translation, and promote their standardisation, validation, and ethical deployment within clinical trials rather than ad hoc adoption.

### 9.9 Global Data Sharing and Registries

- WHO and IXA should establish and maintain a comprehensive, independent international xenotransplantation registry, building on the existing IXA/WHO inventory, to capture longitudinal scientific, safety, and outcome data across jurisdictions and enhance transparency and public trust.

### 9.10 Governance, Capacity Building, and Equity

- WHO and IXA should strengthen international governance through coordinated oversight frameworks, regional capacity‑building initiatives, and regulatory mentorship, with particular attention to equitable participation by low‑ and middle‑income countries.
- Equity considerations should apply throughout the translational pathway, including fair access to clinical trials, avoidance of unregulated practices, and alignment with the principles articulated in the Santander Statement.

### 9.11 Public Engagement and Trust

- WHO and IXA should jointly promote proactive, transparent public engagement, including responsible communication following clinical cases, inclusion of patient and community perspectives, and sustained efforts to counter misinformation and sensationalism.

### 9.12 Continuous Review and Update of Guidance

- IXA, in collaboration with WHO, should commit to regular review and updating of xenotransplantation guidance, ensuring that ethical, scientific, and regulatory frameworks remain aligned with evolving evidence, technologies, and societal expectations.

## 10 Discussion/Conclusion

Collectively, the deliberations of Working Groups 1 through 7 reaffirm that clinical xenotransplantation has transitioned into a phase of cautious, early‑stage human application that demands coordinated scientific, ethical, and regulatory discipline. Advances in genetically engineered porcine source animals (Working Group 1), increasingly standardised clinical trial frameworks (Working Group 2), and evolving immunosuppressive and biomarker‑guided patient‑management strategies (Working Group 3) have together enabled initial clinical feasibility while simultaneously highlighting persistent, context‑dependent immunologic and infectious risks. These risks, particularly those related to donor‑derived porcine pathogens (Working Group 4), reinforce the foundational Changsha principle that biosafety, lifelong surveillance, and transparency are intrinsic, rather than ancillary to ethical clinical conduct (Working Group 5). As xenotransplantation activity expands across jurisdictions, the need for harmonised international governance, shared registries, and coordinated oversight (Working Group 6) becomes increasingly critical to safeguard public trust and scientific integrity. Emerging digital, molecular, and AI‑enabled technologies (Working Group 7) provide powerful tools to integrate donor design, patient selection, monitoring, and adaptive trial management, but derive their legitimacy and value only when embedded within disciplined ethical frameworks and globally aligned governance structures. Taken together, these perspectives converge on a shared conclusion: responsible clinical translation of xenotransplantation requires incremental progress guided by minimum effective standards, rigorous oversight, and sustained international cooperation.

## Funding

The authors have nothing to report.

## Conflicts of Interest

Wayne J. Hawthorne, Peter J. Cowan, Ik Jin Yun, Greg Korbutt, Daniel J. Hurst, Richard N. Pierson III, Robert Rieben, Manuel Pascual, Nicole Scholes‐Robertson, Wei Wang, Emanuele Cozzi, Raphael P.H. Meier, Linda Scobie, Burcin Ekser, Alex Loupy, Hidetaka Hara declare that they have no conflicts of interest to declare. Eckhard Wolf and Paolo Brenner are co‐founders of XTransplant GmbH, Starnberg, Germany. Megan Sykes has received sponsored research funding from United Therapeutics and Choironex/Nefro Health. Léo H. Bühler is a consultant for Clonorgan. Linda Scobie holds research grants with Axiom Xenotherapeutics as Site or Overall Principal Investigator, and serves as a consultant for Axiom PLC. Olga Garkavenko is Founder Director, CSO, NZeno Ltd, New Zealand. Hyunil Kim is a Director of OPTIPHARM Co.,Ltd. Adrian Abalovich is Vice President of Crofabiotech, SA. Rita Bottino is an employee of Imagine Pharma. Muhammad M. Mohiuddin received research grant support from United Therapeutics, Inc., serving as Site or Overall Principal Investigator. Jay Fishman serves as a consultant to eGenesis, Vertex, Eledon, and Makana.

## Data Availability

Data sharing not applicable to this article as no datasets were generated or analysed during the current study.
